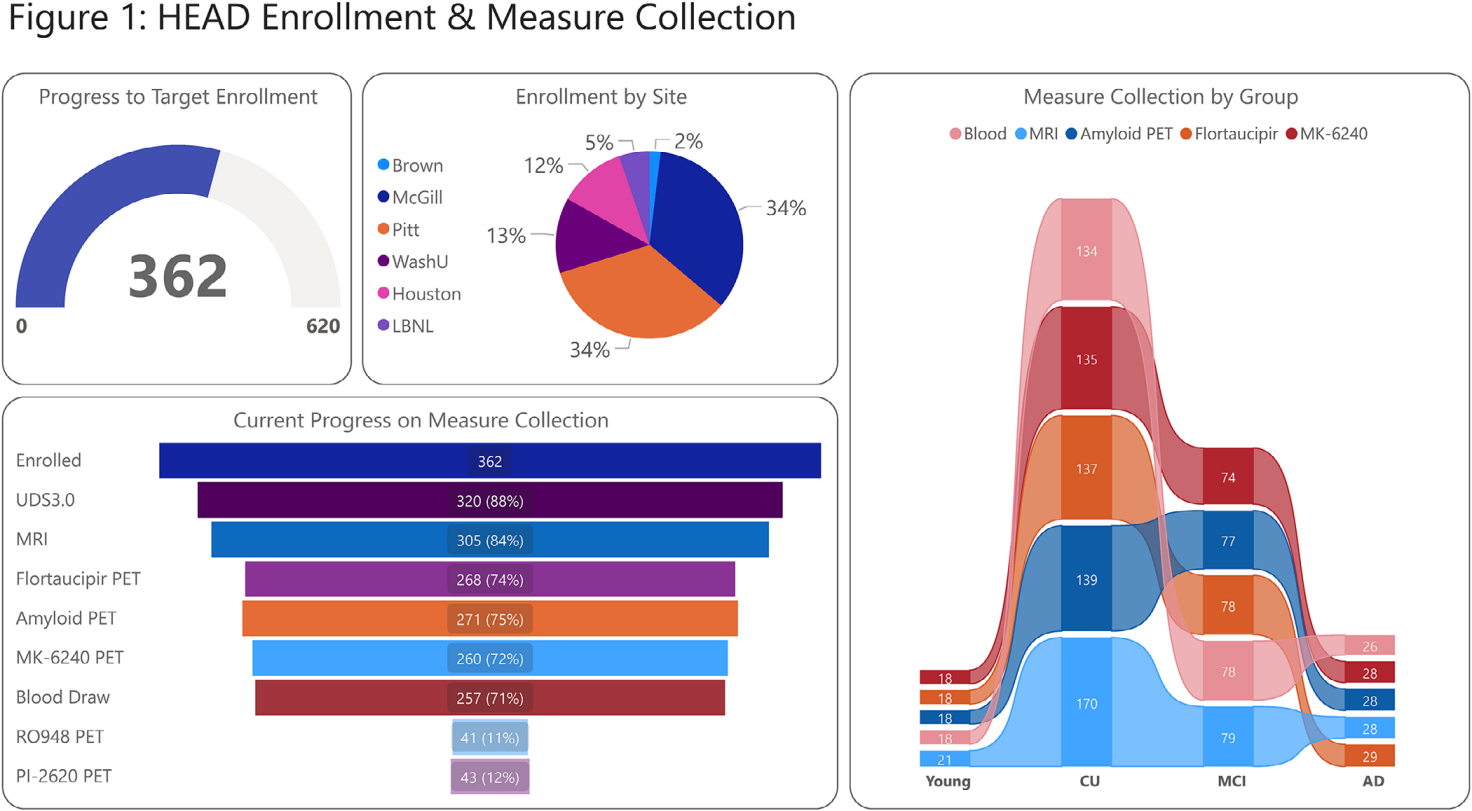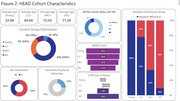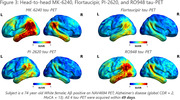# Longitudinal multicenter head‐to‐head harmonization of tau‐PET tracers: an overview of the HEAD study cohort

**DOI:** 10.1002/alz.091910

**Published:** 2025-01-09

**Authors:** Firoza Z Lussier, Guilherme Povala, Guilherme Bauer‐Negrini, Livia Amaral, Juli Cehula, Devin J Fine, Peter Lemaire, Madeleine Bloomquist, Belen Pascual, Brian A. Gordon, Val J. Lowe, Hwamee Oh, David N. soleimani‐meigooni, Pedro Rosa‐Neto, Dana Tudorascu, William J. Jagust, William E Klunk, Suzanne L. Baker, Tharick Ali Pascoal

**Affiliations:** ^1^ University of Pittsburgh, Pittsburgh, PA USA; ^2^ Houston Methodist Research Institute, Houston, TX USA; ^3^ Washington University in St. Louis School of Medicine, St. Louis, MO USA; ^4^ Department of Radiology, Mayo Clinic, Rochester, MN USA; ^5^ Brown University, Providence, RI USA; ^6^ Memory and Aging Center, Weill Institute for Neurosciences, University of California, San Francisco, San Francisco, CA USA; ^7^ Translational Neuroimaging Laboratory, Montréal, QC Canada; ^8^ University of California, Berkeley, Berkeley, CA USA; ^9^ Lawrence Berkeley National Laboratory, Berkeley, CA USA

## Abstract

**Background:**

Standardizing tau pathology quantification in vivo is challenged by differences in binding characteristics between tau‐PET tracers. The HEAD study aims to generate a leading, longitudinal head‐to‐head dataset of MK‐6240, Flortaucipir, RO948, and PI‐2620 tau‐PET to harmonize these tracers' outcomes and develop tools allowing for the generalization of findings across large studies and trials. Here, we present current advancements in building the HEAD study cohort and dataset.

**Methods:**

The HEAD study is managed at the University of Pittsburgh. HEAD comprises several sites across the US and Canada in which 620 subjects (young, cognitively unimpaired, mild cognitive impairment, and Alzheimer’s disease (AD)) will undergo tau‐PET with at least two tracers, amyloid‐PET with PiB or NAV4694, MRI, blood collection, and standardized neuropsychological testing at baseline and at 18‐month follow‐up. PET and MRI acquisition parameters are based on ADNI4 protocols and neuropsychological testing employs the NACC Uniform Data Set. The National Centralized Repository for AD serves as the biorepository for blood samples and the Laboratory of Neuroimaging provides a centralized database for imaging and neuropsychological archiving. PET data is reconstructed to maximize cross‐scanner harmonization and is processed uniformly similarly to ADNI4 PET.

**Results:**

In one year of active enrollment (Jan 2023‐Jan 2024), 362 participants were enrolled at six active sites; over 80% of participants completed neuropsychological testing and MRI, and over 70% completed Flortaucipir, MK‐6240, and amyloid‐PET, with a mean tau‐PET acquisition window of 29.1 days. RO948 and PI‐2620 tau‐PET are additionally acquired in a subset currently including 41 participants (Figure 1). Current HEAD cohort demographics including age, sex, underrepresented populations, and group distributions, APOEε4 carrier status, and amyloid positivity are shown in Figure 2. A case of a subject with AD with all 4 tau‐PET tracers acquired head‐to‐head within 49 days is shown in Figure 3.

**Conclusion:**

The HEAD study represents a continued effort in the optimization of AD imaging biomarkers. Baseline data collection is projected to be completed in 2024; results being generated by multiple groups from this dataset will provide novel and crucial guidance on the use of tau‐PET tracers in research, clinical trials, and prospectively in clinical practice.